# Silica-titania xerogel doped with Mo,P-heteropoly compounds for solid phase spectrophotometric determination of ascorbic acid in fruit juices, pharmaceuticals, and synthetic urine

**DOI:** 10.1186/s13065-016-0233-5

**Published:** 2017-01-03

**Authors:** Maria A. Morosanova, Elena I. Morosanova

**Affiliations:** Analytical Chemistry Division, Chemistry Department, Lomonosov Moscow State University, Moscow, Russia

**Keywords:** Silica-titania xerogel, Heteropoly compounds, Ascorbic acid, Solid phase spectrophotometry, Food analysis, Pharmaceutical analysis, Biological liquids analysis

## Abstract

**Background:**

Ascorbic acid is one of the most important vitamins to monitor in dietary sources (juices and vitamins) and biological liquids.

**Results:**

Silica and silica-titania xerogels doped with Mo,P-heteropoly compounds (HPC) have been synthesized varying titanium(IV) and HPC content in sol. Their surface area and porosity have been studied with nitrogen adsorption and scanning electron microscopy, their elemental composition has been studied with energy-dispersive X-ray analysis. The redox properties of the sensor material with sufficient porosity and maximal HPC content have been studied with potentiometry and solid phase spectrophotometry and it has been used for solid phase spectrophotometric determination of ascorbic acid. The proposed method is characterized by good selectivity, simple probe pretreatment and broad analytical range (2–200 mg/L, LOD 0.7 mg/L) and has been applied to the analysis of fruit juices, vitamin tablets, and synthetic urine.

**Conclusions:**

New sensor material has been used for simple and selective solid phase spectrophotometric procedure of ascorbic acid determination in fruit juices, vitamin tablets, and synthetic urine.

**Graphical abstract:**
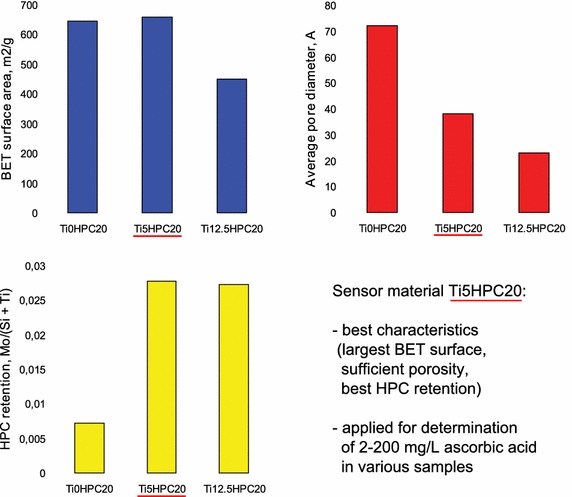
We synthesized several silica-titania xerogels doped with Mo,P-heteropoly compounds, studied their properties, and designed the sensor material for solid phase spectrophotometric determination of ascorbic acid in fruit juices, pharmaceuticals, and synthetic urine.

## Background

Ascorbic acid (AA) is one of the most important vitamins in human diet; it is involved in various biochemical pathways and acts as a powerful antioxidant [1]. It is important to monitor AA levels in dietary sources (fruits and vegetables, food supplements, vitamin formulations) as well as in biological liquids, such as urine and serum.

Methods for AA determination include redox titration with 2,6-dichlorophenolindophenol (DCPIP) [2], HPLC [3], electrochemical [4, 5], fluorescent and spectrophotometric methods [6–16].

Spectrophotometric methods are the most frequently applied ones, due to their simplicity. The direct spectrophotometric determination of AA by its absorption in the ultraviolet region is very difficult because of many interfering substances, usually present in the AA containing samples [16]. This problem is overcome by the use of reagents which have specific color reactions with AA. Most of these methods are based on the AA reducing ability and thus, use the metal colored complexes (Fe(III)-ferrozine [11], Fe(III)-1,10-phenantroline [12], Cu(II)-neocuproine [13]) or the heteropoly compounds (HPC) [14, 15, 17] as chromogenic reagents. The reducing ability of AA can also be employed in fluorescent determination, as AA reduces copper(II) to copper(I) which releases graphene quantum dots fluorescence [6].

The immobilization of HPC seems to be a promising approach to obtain the sensor materials for AA determination. The presence of metal ions [Cu(II), Ti(IV), Bi(III)] in solution has been previously shown to accelerate the HPC reduction, both in solution and in solid phase. Silica xerogels doped with HPC and copper(II) have been used for AA determination in soft drinks [17]. The use of mixed silica-titania xerogels may offer new possibilities for the sensor material development. The development of simple methods of AA determination in complex samples is an important task.

The aim of the present work was to develop the synthesis method of silica-titania xerogel doped with Mo,P-HPC and use it as a sensor material for the solid phase spectrophotometric determination of ascorbic acid in fruit juices, complex vitamin formulations and synthetic urine.

## Experimental

### Reagents and apparatus

The following reagents were purchased from Acros Organics: calcium chloride, magnesium chloride, sodium chloride, potassium chloride, ammonium chloride, sodium sulfate, trisodium citrate, sodium oxalate, potassium phosphate, sodium phosphate, ascorbic acid, glucose, creatinine, urea, 2,6-dichlorophenolindophenol, hydrochloric acid, nitric acid, molybdenum(VI) oxide, titanium(IV) tetraethoxyde, and tetraethyl orthosilicate. All the reagents were of analytical grade, titanium(IV) tetraethoxyde was of technical grade.

Mo,P-HPC (Vavele reagent) was prepared as following: 7.0 g of MoO_3_, 14.0 g of sodium carbonate and 2.0 g of sodium phosphate were mixed with 20.0 mL of nitric acid and then the volume was adjusted to 100.0 mL.

Stock solutions of ascorbic acid were prepared with doubly distilled water and only freshly prepared solutions were used.

Silica-titania xerogels were obtained by drying in Ethos microwave equipment (Milestone, Italy). Surface area, porosity BET analysis, and BJH pore distribution analysis were carried out with ASAP 2000 (Micromeritics, USA). Scanning electron microscopy (SEM) images were collected with the use of LEO Supra 50 VP (Zeiss, Germany) operating at 20 kV, analysis was performed under 40 Pa of nitrogen to reduce charging effects. Energy-dispersive X-ray analysis (EDX) was performed using X-MAX 80 spectrometer (Oxford Instruments, UK): analysis was performed in the VP mode at 20 kV with 60 µm aperture, the distance to the sample was 12 mm. The elemental composition was calculated using INCA software (Oxford Instruments, UK). Absorbance of the xerogels water suspensions was measured using Lambda 35 spectrophotometer (PerkinElmer, USA) equipped with 50 mm integrating sphere (Labsphere, USA), l 1.0 mm.

### Synthesis of silica and silica-titania xerogels

Xerogels were obtained as following: 10.00 mL of solution with various content of Mo,P-HPC in and 10.00 mL of ethanol were added to 10.00 mL of the precursors mixture, containing from 0 to 12.5% titanium(IV) tetraethoxyde and from 100 to 87.5% tetraethoxysilane, while stirring (Table 1). The wet gel was formed in the next 72 h. The wet gels were dried at 800 W microwave irradiation for 10 min to get dry xerogels. Xerogels were washed with distilled water and 1.0 mol/L hydrochloric acid.

Five xerogels have been synthesized, their names and composition are given in Table 1.

**Table 1:** The composition of sol and names for sol–gel materials synthesized in present work

	Ti12.5HPC0	Ti0HPC20	Ti5HPC20	Ti12.5HPC20	Ti5HPC30
Tetraethyl orthosilicate, mL	8.75	10.00	9.95	8.75	9.95
Titanium(IV) tetraethoxyde, mL	1.25	–	0.05	1.25	0.05
Ethanol, mL	10.00	10.00	10.00	10.00	10.00
Mo,P-HPC, mL	–	6.00	6.00	6.00	9.00
0.05 mol/L HCl, mL	10.00	4.00	4.00	4.00	1.00

### General procedure for the ascorbic acid—silica-titania xerogels interaction study

0.10 g of silica-titania xerogel was added to 5.0 mL of solution, containing 50 mg/L of ascorbic acid at different pH and the obtained mixture was shaken for 2–30 min. Then the xerogels absorbance was measured at 740 nm. The optimal conditions were chosen in order to reach the maximal absorbance.

### Sample preparation and solid phase spectrophotometric determination procedure

Vitamin tablets samples were prepared as following: One tablet was dissolved in the distilled water and the volume was adjusted to 100.0 mL.

Fruit juices were diluted by 5 times with the distilled water.

Synthetic urine was prepared as in [18] and consisted of CaCl_2_ (0.65 g/L), MgCl_2_ (0.65 g/L), NaCl (4.6 g/L), Na_2_SO_4_ (2.3 g/L), trisodium citrate (0.65 g/L), sodium oxalate (0.02 g/L), KH_2_PO_4_ (2.8 g/L), KCl (1.6 g/L), NH_4_Cl (1.0 g/L), urea (25.0 g/L), creatinine (1.1 g/L), and 2% (wt/vol) glucose.

0.10 g of silica-titania xerogel and 0.5 mL of acetate buffer (pH 4.0) were added to 5.0 mL of the treated sample solution, the obtained mixture was shaken for 20 min, then the xerogels absorbance was measured at 740 nm. The concentration of ascorbic acid in the sample was calculated using the calibration curve. For the latter the absorbance of the xerogels after interaction with the standard solutions of ascorbic acid in the range of 2–200 mg/L was measured. Least squares method was used to obtain the calibration curve.

## Results and discussions

### The synthesis of the xerogels doped with hetepolycompounds and their characterization

Heteropoly compounds (HPC) are widely used analytical reagents. Silica xerogels doped with various HPC have been synthesized earlier and applied as sensor [17] or catalytic materials [19, 20]. The Mo,P-HPC incorporated in the silica xerogel have retained their redox properties and the obtained sensor material has been applied to AA determination [17]. Tungstophosphoric and molypdophosphoric heteropoly acids have been used for the creation of photocatalytic titania sol–gel materials [21–23]. The effect of photocatalytic materials modification with HPC is the increase of catalytic activity and the conductivity of the materials [19–23]. Silica-titania materials doped with HPC have not been synthesized before.

The textural characteristics of the xerogels are very important for the analytical application, so in the present work the influence of HPC and titanium(IV) content on the textural characteristics of silica-titania xerogels was investigated. Another important characteristic is the amount or retained HPC and its redox properties, as it is crucial for the redox properties of the modified xerogel itself.

**Fig. 1:**
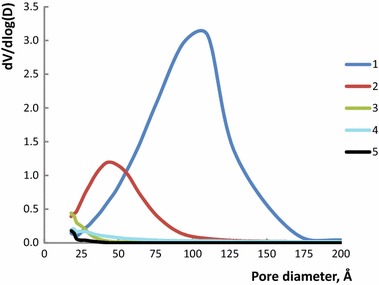
Pore size distributions according to the BJH method calculated from the desorption branches for xerogels doped with HPC.* 1* Ti0HPC20,* 2* Ti5HPC20,* 3* Ti12.5HPC20,* 4* Ti5HPC30,* 5* Ti12.5HPC0 (see Table 1 for xerogel names)

**Fig. 2:**
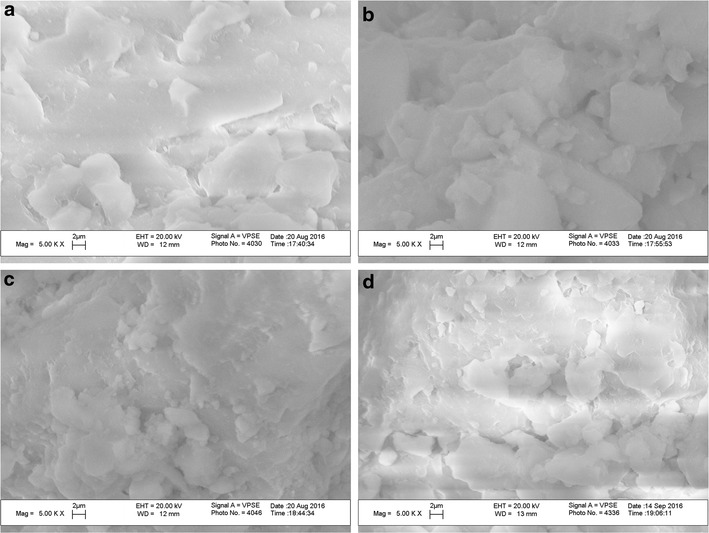
SEM images of the xerogels surface: **a** Ti12.5HPC0, **b** Ti0HPC20, **c** Ti5HPC20, **d** Ti12.5HPC20 (see Table 1 for xerogel names)

We varied the titanium(IV) and HPC content in the mixed xerogels to obtain the best suited sensor material. The modified silica and silica-titania xerogels were characterized by yellow color indicating the presence of HPC. The textural characteristics of the studied xerogels have been investigated using nitrogen adsorption; the data are given in Table 2. The significant decrease in average pore diameter, total pore volume and BET surface is observed with the increase of titanium(IV) content, similarly to what has been observed for titania sol–gel materials [21]. The attempt to increase the HPC content (xerogel Ti5HPC30) also led to the significant decrease in the same characteristics, further proving the great influence of HPC/Ti ratio. The average pore size for Ti5HPC30 and Ti12.5HPC20 were similar to the unmodified silica-titania xerogel (Ti12.5HPC0). The pores distribution according to the BJH method for the studied xerogels is given in Fig. 1. These data demonstrate the differences in the materials porosity, that are also observed on SEM images (Fig. 2).

**Table 2:** The textural properties of xerogels

	Ti0HPC20	Ti5HPC20	Ti12.5HPC20	Ti5HPC30	Ti12.5HPC0
BET surface area, m^2^/g	644	658	450	220	454
Total pore volume, cm^3^/g	1.1	0.6	0.2	0.14	0.21
Average pore diameter, Å	72	38	23	25	18.5

This can be explained by the direct interaction of titanium(IV) with HPC which interferes with gel formation cross-linking interactions. The influence of HPC on the titania sol–gel materials textural results in the decrease in BET surface area and average pore diameter with the increase of the HPC content [21, 23].

**Fig. 3:**
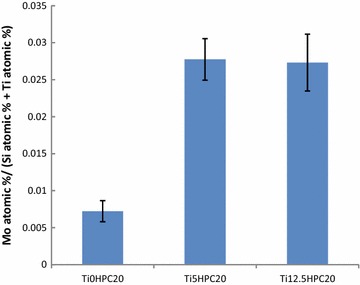
The ratio of Mo atomic percentage to the sum of Si and Ti atomic percentages for three modified xerogels (n = 10, P = 0.95) (see Table 1 for xerogel names)

EDX analysis allowed determination of the elemental composition of the xerogels Ti12.5HPC0, Ti0HPC20, Ti5HPC20, and Ti12.5HPC20. The titanium content values are given in Table 3. No molybdenum content was determined in the Ti12.5HPC0 sample, and the molybdenum content ratio to the matrix (the sum of Si and Ti content) for other xerogels is shown in the Fig. 3. The HPC is much better retained in the silica-titania xerogels than in silica xerogel, but the increase of titanium content shows no further increasing effect.

**Table 3:** The titanium content (Ti/(Ti + Si)) in xerogels according to the energy-dispersive X-ray analysis (n = 10, P = 0.95)

Xerogel sample	Predicted Ti content, %	Ti content found by EDX, %
Ti0HPC20	0	Not found
Ti5HPC20	5	4.4 ± 0.7
Ti12.5HPC20	12.5	13.6 ± 2.6
Ti12.5HPC0	12.5	13.5 ± 1.4

In our previous works the importance of larger pores for the sensor material properties has been shown [24, 25]. Considering the combination of high HPC content and rather large pores, the Ti5HPC20 was selected for study in further experiments.

The redox potential (ORP) of the HPC immobilized in this xerogel was measured: the ORP of the immobilized reagent is usually defined as the potential, measured in the mediator solution [26]. To study the ORP the series of mixed K_3_[Fe(CN)_6_] and K_4_[Fe(CN)_6_] 2 × 10^−3^ mol/L solutions (the ratio varied from 1:100 to 100:1) were used as proposed earlier [27]. After the interaction the ORP of the solution and the xerogel absorbance were measured. The absorbance measurements (A) allowed calculation of the concentration of the reduced blue HPC (C_red_) in the solid phase. The concentration of the oxidized HPC (C_ox_) was calculated using the difference between A_max_ and A, where A is the absorbance of the xerogel and A_max_ is the absorbance of the xerogel after the interaction with the excess of K_4_[Fe(CN)_6_] (2 × 10^−2^ mol/L). The Nernst equation for this system is the following:

$$E = E^{{0}{\prime}} + \frac{0.058}{2} \cdot \lg \frac{{C_{ox} }}{{C_{red} }} = E^{{0}{\prime}} + \frac{0.058}{2} \cdot \lg \frac{{A_{\text{max} } - A}}{A}$$

The formal ORP value was calculated using the following equation:

$$E^{{0}{\prime}} = E - \frac{0.058}{2} \cdot \lg \frac{{A_{\hbox{max} } - A}}{A}$$

Simple linear regression analysis of the experimental data allowed calculating E^0^ʹ = 0.26 V (Pt electrode vs Ag/AgCl electrode). The ORP of Mo,P-HPC varies between 0.36 and 0.46 V, depending on their composition [28]. Similar decrease in ORP due to immobilization has been shown previously for redox indicator DCPIP [26]. The decrease of immobilized HPC ORP may lead to more selective response of sensor material to AA in the presence of weaker reducing agents, e.g. polyphenols.

Basing on the studied characteristics (porosity, elemental composition, redox potential) Ti5HPC20 was selected as sensor material for solid phase spectrophotometric AA determination.

### Interaction of xerogels doped with HPC with ascorbic acid and analytical application

**Fig. 4:**
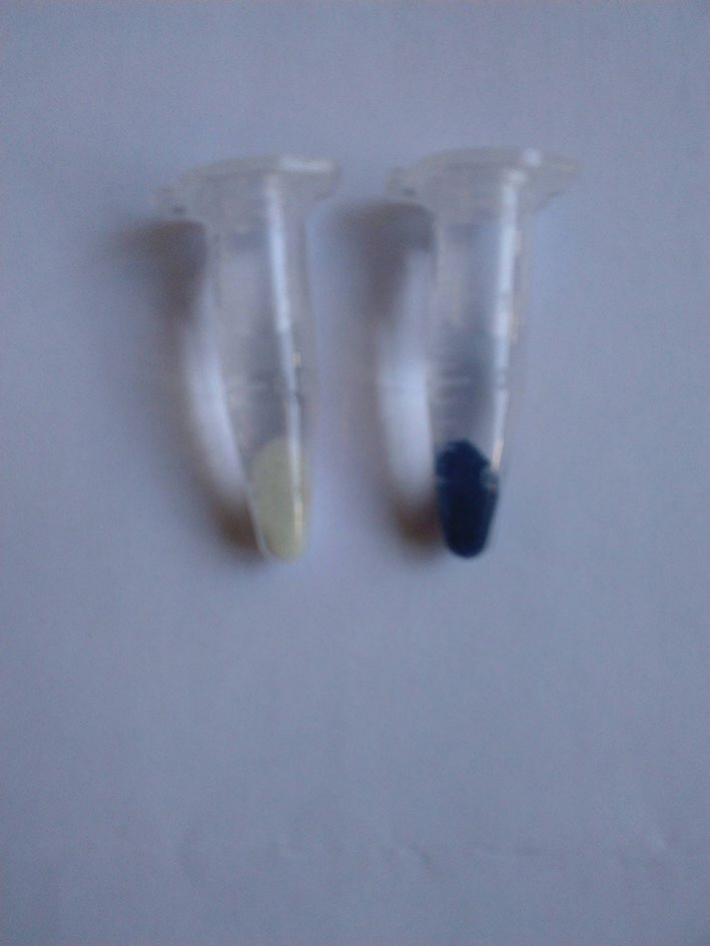
The coloration of Ti5HPC20 before (*left*) and after (*right*) interaction with 200 mg/L of ascorbic acid (see Table 1 for xerogel names)

The interaction of silica xerogels doped with HPC with reducing agents leads to the formation of blue HPC coloring [17]. The contact of silica-titania xerogel doped with HPC (Ti5HPC20) with ascorbic acid resulted in the xerogels color change from pale yellow to dark blue (Fig. 4). The UV–vis absorption spectrum of colored xerogel, obtained by this reaction, showed the maximum at 740 nm. The interaction of ascorbic acid with xerogel incorporated titanium(IV), reported earlier [29], does not interfere, as it has the absorbance maximum at 390 nm.

The medium acidity influence on the interaction was studied in the range of 1.5–9.0. The maximal values of the xerogels absorbance were observed at pH 4.0–6.0. As polyphenols antioxidant activity increases and ORP decreases with the increase of pH (as shown for catechol [30]), the pH 4.0 was chosen to minimize possible interferences.

The kinetics of Ti5HPC20 xerogels interaction with ascorbic acid was studied. Comparing the results with the previously obtained data on the silica xerogels doped with HPC [17] we observed significant increase in the speed of interaction with 20 mg/L of AA:

**Table:** 

Xerogel	Equilibrium time, min	Half-reaction period, min	Reference
Silica doped with HPC	100	40	[17]
Ti5HPC20	20	9.6	Present work

Titanium(IV) incorporated in the matrix of sensor material accelerates the reduction of immobilized HPC, as titanium(IV) ions accelerated the reduction of HPC in the solution [17].

**Fig. 5:**
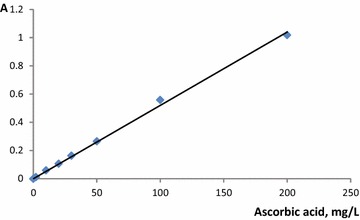
Calibration curve of solid phase spectrophotometric ascorbic acid determination using Ti5HPC20 (see Table 1 for xerogel names). m(Ti5HPC20) = 0.10 g, V = 5.0 mL, pH 4.0, time of contact 20 min

To develop the analytical procedure for solid phase spectrophotometric determination of ascorbic acid the calibration curve was constructed: the analytical range was 2–200 mg/L (A = 0.0052∙C, R^2^ = 0.9976, Fig. 5). The limit of detection was calculated as 3∙standard deviation of the blank divided by the slope value and equaled 0.7 mg/L (n = 3, P = 0.95). This procedure was applied to the AA determination in fruit juices, vitamin tablets and spiked synthetic urine sample.

#### Fruit juices

Ascorbic acid is usually present in a large variety of commercial fruit juices. AA determination is necessary to monitor the quality of fruit juices. The interference of polyphenols, naturally occurring in fruit juices along with AA, on the AA interaction with Ti5HPC20 was investigated. In the previous work silica-titania xerogels have been shown to interact with polyphenols and AA, which has resulted in yellow coloration of the xerogel [29]. The complex forming reaction is not selective, so silica-titania xerogel without immobilized HPC can only be used for AA determination in simple objects.

The polyphenols did not interfere the reducing of HPC in the presence of 10 mg/L AA in the substantial amounts (Table 4). The selectivity of Ti5HPC20 was higher when compared to the unmodified silica-titania xerogel for all studied interferences. This describes HPC immobilization in silica-titania xerogel as a promising approach for the AA determination in the presence of polyphenols.

**Table 4:** The results of solid phase spectrophotometric AA determination (10 mg/L) in the presence of polyphenols

Polyphenol	Interference threshold, mg/L	
	Silica-titania xerogel (Ti12.5HPC20)	Silica-titania xerogel doped with HPC (Ti5HPC20)
Taxifolin	<0.5	50
Dopamine	<0.5	50
Caffeic acid	<0.5	50
Gallic acid	<0.5	100
Quercetin	100	200
Rutin	100	250
Ferulic acid	100	250
Catechol	<0.5	50

Sulfites are widely used as preservatives in food and drinks. The influence of sulfite on the AA determination was investigated. Sulfite concentrations above 20 mg/L interfered AA determination. Orange juice was reported to contain 50–100 mg/L of sulfites (63 mg/L [31], 104 mg/L [32]), so with the dilution of samples used in the proposed procedure sulfites cannot influence AA determination.

Ascorbic acid content was determined using the calibration curve and the results of determination were compared with DCPIP titrimetric determination (Table 5). There was a good agreement between these two methods, except the case of blood orange juice, where the red color of the sample did not allow establishing the titration end point (which should be pink).

**Table 5:** Solid spectrophotometric determination of ascorbic acid in various samples (n = 3, P = 0.95)

Sample (AA declared content)	Found with solid phase spectrophotometry (RSD, %)	Found with titrimetric method (RSD, %)
Synthetic urine spiked with 20 mg L^−1^ of ascorbic acid	20.46 ± 2.34 mg L^−1^ (6.7)	19.58 ± 0.81 mg L^−1^ (2.4)
Naturetto vitamin tablets (7 mg/tablet)	7.29 ± 0.81 mg/tablet (6.5)	7.00 ± 0.20 mg/tablet (1.7)
Naturino vitamin tablets (11.25 mg/tablet)	10.43 ± 0.95 mg/tablet (5.3)	10.73 ± 0.55 mg/tablet (3.0)
Orange juice (200 mg L^−1^)	179.30 ± 21.50 mg L^−1^ (7.0)	175.00 ± 10.66 mg L^−1^ (3.6)
Blood orange juice (90 mg L^−1^)	101.67 ± 12.73 mg L^−1^ (7.3)	ND^a^

^a^Not determined, due to red color of the sample interfering with establishing titration endpoint

#### Vitamin tablets

Ascorbic acid acid is widely used in the pharmaceutical formulations, both as the vitamin and as the antioxidant. Two vitamin tablets were analyzed in the present work: Naturetto (glucose—2250 mg, ascorbic acid—7 mg, vitamin E—1.5 mg) and Naturino (biotin—6.3 mg, vitamin B1—0.18 mg, vitamin B2—0.227 mg, vitamin B12—0.37 mg, ascorbic acid—11.25 mg, vitamin E—1.87 mg). The results of vitamin tablets analysis are given in the Table 5. The vitamin tablets composition did not influence the determination, as the results of the analysis agreed with the standard method.

#### Synthetic urine sample

Ascorbic acid is a chemical substance with significant role in human and its determination is highly desirable for analytical and diagnostic applications. The AA content in urine corresponds with its content in the serum, and is a valuable marker for the non-invasive analysis [16].

The results of recovery test of solid phase spectrophotometric determination of ascorbic acid synthetic urine are given in Table 6. The recoveries were found to be in the range 96.3–102.7% which describes the proposed procedure reliable for the urine analysis. The comparison of AA determination in the spiked sample with the proposed and DCPIP methods is given in Table 5.

**Table 6:** Recovery test of solid phase spectrophotometric determination of ascorbic acid in the synthetic urine (n = 3, P = 0.95)

Added, mg/L	Found, mg/L	RSD, %	Recovery, %
20.0	20.54 ± 2.56	7.3	102.7
30.0	28.88 ± 1.17	2.4	96.3
50.0	49.76 ± 1.47	1.7	99.5

The analytical application results show many various possibilities of using the created sensor material. The selectivity, sensitivity and rapidity of the proposed method make it suitable for food quality control, pharmaceutical and biological analysis.

## Conclusion

The solid phase spectrophotometric method of ascorbic acid determination has been proposed using the silica-titania xerogel doped with HPC. The influence of titanium(IV) and HPC content on the xerogel properties have been investigated, and the xerogel with relatively big pores and maximal HPC content has been chosen for the AA determination. The analytical range of 2–200 mg/L is broader than the ranges described in literature (Table 7), which is important as AA is a very abundant substance. The proposed method has been characterized by good selectivity and simple probe treatment. The procedures for solid phase spectrophotometric AA determination in fruit juices, vitamin tablets and synthetic urine have been proposed and the results of determination have been shown to be in good agreement with standard method results.

**Table 7:** Comparison of the analytical ranges of various spectrophotometric methods of AA determination

Method	Reagent or sensor material	Linear range, mg/L	Reference
Spectrophotometry	2,6-Dichloroindophenol	1–20	[7]
	*p*-Aminobenzoic acid	20–67	[8]
	Diazotized 1-aminoanthraquinone	5–25	[9]
	Fe(III)- 2,4,6-tripyridyl-s-triazine	2–40	[10]
	Fe(III)-ferrozine	0.2–10	[11]
	Fe(III) 1,10-phenantroline	1–80	[12]
	Cu(II)-neocuproine	1.4–14	[13]
	V,W,P-HPC	1–80	[14]
	V,W,P-HPC	2–24	[15]
	UV absorbance	2.5–30	[16]
Solid phase spectrophotometry	Sephadex QAE A-25, UV absorbance	0.3–5.0	[16]
	Silica xerogel doped with Mo,P-HPC	1–50	[17]
	Bindschedler’s Green immobilized on SiO_2_–SO_3_H	0.3–5	[27]
	Silica-titania xerogel	6–110	[29]
	Silica-titania xerogel doped with Mo,P-HPC	2–200	Present work
